# (*E*)-3-(4-Hy­droxy-3-meth­oxy­phen­yl)-1-(4-hy­droxy­phen­yl)prop-2-en-1-one

**DOI:** 10.1107/S1600536814008757

**Published:** 2014-04-26

**Authors:** S. Sathya, D. Reuben Jonathan, K. Prathebha, G. Usha, J. Jovita

**Affiliations:** a PG and Research Department of Physics, Queen Mary’s College, Chennai-4, Tamilnadu, India; b PG and Research Department of Chemistry, Presidency College, Chennai-5, Tamil Nadu, India

## Abstract

In the title compound, C_16_H_14_O_4_, there is an intra­molecular O—H⋯O hydrogen bond. The benzene rings are inclined to one another by 13.89 (9)°. The prop-2-en-1-one group is twisted slightly, the O=C—C_ar_—C_ar_ (ar = aromatic) and C=C—C=O torsion angles being −10.4 (3) and −7.4 (3)°, respectively. In the crystal, mol­ecules are linked by O—H⋯O hydrogen bonds, forming chains along [100]. These chains are further linked by O—H⋯O hydrogen bonds, forming corrugated sheets lying parallel to (010). There are C—H⋯π inter­actions present within the sheets.

## Related literature   

For the biological activity of chalcones and chalcone derivatives, see: Marais *et al.* (2005 ▶); Di Carlo *et al.* (1999 ▶); Troeberg *et al.* (2000 ▶); Ni *et al.* (2004 ▶). For a related structure, see: Jasinski *et al.* (2011 ▶). For the synthesis, see: Sidharthan *et al.* (2012 ▶); Chitra *et al.* (2013 ▶). For standard bond lengths, see: Allen *et al.* (1987 ▶).
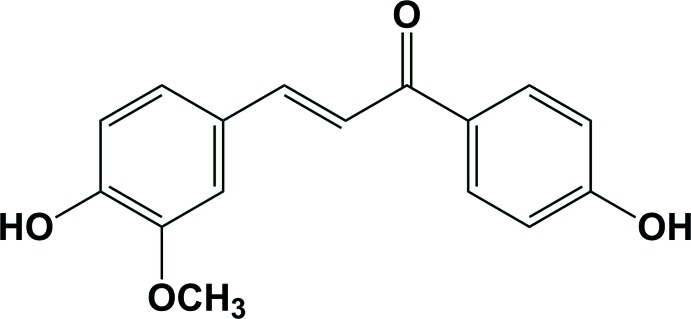


## Experimental   

### 

#### Crystal data  

C_16_H_14_O_4_
*M* *_r_* = 270.27
Orthorhombic, 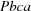

*a* = 16.2808 (8) Å
*b* = 10.4348 (5) Å
*c* = 16.2905 (7) Å
*V* = 2767.5 (2) Å^3^
*Z* = 8
Mo *K*α radiation
μ = 0.09 mm^−1^
*T* = 293 K
0.35 × 0.30 × 0.25 mm

#### Data collection  

Bruker Kappa APEXII CCD diffractometer
Absorption correction: multi-scan (*SADABS*; Bruker, 2004 ▶) *T*_min_ = 0.968, *T*_max_ = 0.977
12921 measured reflections
3441 independent reflections
1935 reflections with *I* > 2σ(*I*)
*R*_int_ = 0.027

#### Refinement  

*R*[*F* ^2^ > 2σ(*F* ^2^)] = 0.039
*wR*(*F* ^2^) = 0.128
*S* = 1.05
3344 reflections
189 parameters
2 restraints
H atoms treated by a mixture of independent and constrained refinement
Δρ_max_ = 0.18 e Å^−3^
Δρ_min_ = −0.20 e Å^−3^

### 

Data collection: *APEX2* (Bruker, 2004 ▶); cell refinement: *APEX2* and *SAINT* (Bruker, 2004 ▶); data reduction: *SAINT* and *XPREP* (Bruker, 2004 ▶); program(s) used to solve structure: *SIR92* (Altomare *et al.*, 1993 ▶); program(s) used to refine structure: *SHELXL97* (Sheldrick, 2008 ▶); molecular graphics: *ORTEP-3 for Windows* (Farrugia, 2012 ▶); software used to prepare material for publication: *SHELXL97* and *PLATON* (Spek, 2009 ▶).

## Supplementary Material

Crystal structure: contains datablock(s) I, New_Global_Publ_Block. DOI: 10.1107/S1600536814008757/su2713sup1.cif

Structure factors: contains datablock(s) I. DOI: 10.1107/S1600536814008757/su2713Isup2.hkl

Supporting information file. DOI: 10.1107/S1600536814008757/su2713Isup3.cml

CCDC reference: 991699

Additional supporting information:  crystallographic information; 3D view; checkCIF report

## Figures and Tables

**Table 1:** Hydrogen-bond geometry (Å, °) *Cg* is the centroid of the C1–C6 benzene ring.

*D*—H⋯*A*	*D*—H	H⋯*A*	*D*⋯*A*	*D*—H⋯*A*
O4—H4*O*⋯O3	0.86 (2)	2.20 (3)	2.655 (2)	113 (2)
O2—H2*O*⋯O1^i^	0.87 (2)	1.87 (2)	2.7349 (18)	173 (2)
O4—H4*O*⋯O1^ii^	0.86 (2)	2.22 (2)	2.937 (2)	141 (3)
C16—H16*A*⋯*Cg* ^ii^	0.96	2.86	3.747 (3)	155

Symmetry codes: (i) 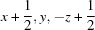; (ii) 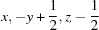.

**Fig. 1.**
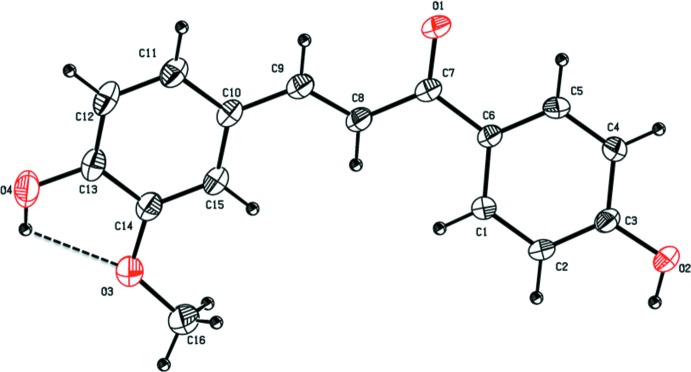
The molecular structure of the title molecule, with atom labelling. Displacement ellipsoids are drawn at the 30% probability level.

**Fig. 2.**
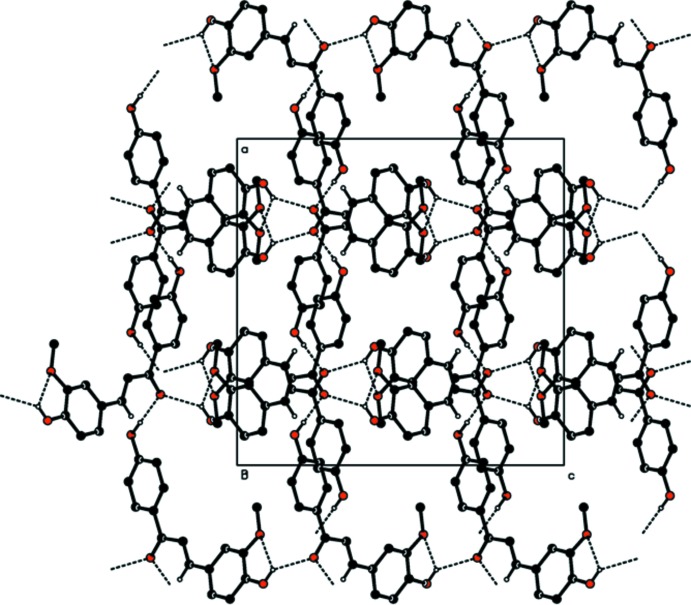
A view along the x axis of the crystal packing of the title compound. Hydrogen bonds are shown as dashed lines (see Table 1 for details).

## supplementary crystallographic information

### 1. Comment

Chalcones are known as the precursors of all flavonoid type natural products in biosynthesis (Marais *et al.*, 2005). They are a major class of natural products with widespread distribution in fruits, vegetables, spices, tea and soy based food stuff and have been the subjects of interest for their significant pharmacological activities (Di Carlo *et al.*, 1999). Many chalcones have been described for their high anti-malarial activity, probably as a result of addition of nucleophilic species to the double bond of the enone (Troeberg *et al.*, 2000). A review of anti-infective and anti-inflammatory chalcones and recent advances in therapeutic chalcones have been reported (Ni *et al.*, 2004). To understand the three dimensional features of this class of compounds, we report herein on the crystal structure of the title compound.

The molecular structure of the title molecule is illustrated in Fig. 1. The bond lengths (Allen *et al.*, 1987) and bond angles are within normal values. The bond angles C6—C7—C8 = 119.12 (14) ° and C8═C9—C10 = 127.20 (17) ° are comparable with those in a similar reported structure (*E*)-3-(3,4-Dimethoxyphenyl)-1-(2-hydroxyphenyl)prop-2-en-1-one (2E)-3-(3,4-Dimethoxyphenyl)-1-(4-hydroxyphenyl)prop-2-en-1-one (Jasinski *et al.*, 2011). This may be due to the presence of the keto group and associated steric forces. The prop-2-en-1-one group is twisted slightly with torsion angles O1═C7—C8—C9 and C5—C6—C7═O1 being -10.4 (3) and -7.4 (3) °, respectively. These values are comparable with the value of -6.9 (2) and -15.9 (2) ° reported for the above mentioned structure.

In the crystal, molecules are linked by O—H···O hydrogen bonds forming chains propagating along the *a* axis (Table 1 and Fig. 2). These chains are linked by further O—H···O hydrogen bonds forming corrugated sheets lying parallel to (010). Within the sheets there are C—H···π interactions present (Table 1). In total each molecule is linked to four neighbours by O—H···O hydrogen bonds (Table 1 and Fig. 2). Atom O4 acts as a bifurcated donor forming intra- and inter-molecular O—H···O hydrogen bonds (Table 1 and Fig. 2).

### 2. Experimental

The title compound was synthesized by a published procedure using the acid catalyzed Claisen-Schmidt reaction (Sidharthan *et al.*, 2012; Chitra *et al.*, 2013) Dry HCl gas was passed through a well cooled and stirred solution of 4-hydroxyacetophenone (0.02 mol) and vanillin (0.02 mol) in 125 ml of dry ethanol, placed in a 250 ml round-bottomed flask, for about 1 h. A wine-red coloured solution was formed to which ice cold water was added. Yellow block-like crystals of the title compound separated and were washed with double distilled water and re-crystallized from hot ethanol (Yield 85%; *M*.p. 454 K).

### 3. Refinement

The OH H atoms were located from difference Fourier maps and refined with *U*_iso_(H)= 1.5*U*_eq_(O). The C-bound H atoms were positioned geometrically and treated as riding atoms: C—H distance of 0.93 - 0.96 Å with *U*_iso_(H)= 1.5*U*_eq_(*C*-methyl) and = 1.2*U*_eq_(C) for other H atom.

### Crystal data

**Table:**

C_16_H_14_O_4_	*F*(000) = 1136
*M**_r_* = 270.27	*D*_x_ = 1.297 Mg m^−^^3^
Orthorhombic, *P**b**c**a*	Mo *K*α radiation, λ = 0.71073 Å
Hall symbol: -P 2ac 2ab	Cell parameters from 3441 reflections
*a* = 16.2808 (8) Å	θ = 2.5–28.3°
*b* = 10.4348 (5) Å	µ = 0.09 mm^−^^1^
*c* = 16.2905 (7) Å	*T* = 293 K
*V* = 2767.5 (2) Å^3^	Block, yellow
*Z* = 8	0.35 × 0.30 × 0.25 mm

### Data collection

**Table:**

Bruker Kappa APEXII CCD diffractometer	3441 independent reflections
Radiation source: fine-focus sealed tube	1935 reflections with *I* > 2σ(*I*)
Graphite monochromator	*R*_int_ = 0.027
ω and φ scan	θ_max_ = 28.3°, θ_min_ = 2.5°
Absorption correction: multi-scan (*SADABS*; Bruker, 2004)	*h* = −21→17
*T*_min_ = 0.968, *T*_max_ = 0.977	*k* = −13→7
12921 measured reflections	*l* = −21→21

### Refinement

**Table:**

Refinement on *F*^2^	Secondary atom site location: difference Fourier map
Least-squares matrix: full	Hydrogen site location: inferred from neighbouring sites
*R*[*F*^2^ > 2σ(*F*^2^)] = 0.039	H atoms treated by a mixture of independent and constrained refinement
*wR*(*F*^2^) = 0.128	*w* = 1/[σ^2^(*F*_o_^2^) + (0.0473*P*)^2^ + 0.8783*P*] where *P* = (*F*_o_^2^ + 2*F*_c_^2^)/3
*S* = 1.05	(Δ/σ)_max_ = 0.004
3344 reflections	Δρ_max_ = 0.18 e Å^−^^3^
189 parameters	Δρ_min_ = −0.20 e Å^−^^3^
2 restraints	Extinction correction: *SHELXL97* (Sheldrick, 2008), Fc^*^=kFc[1+0.001xFc^2^λ^3^/sin(2θ)]^-1/4^
Primary atom site location: structure-invariant direct methods	Extinction coefficient: 0.0024 (6)

### Special details

**Table:**

Geometry. All e.s.d.'s (except the e.s.d. in the dihedral angle between two l.s. planes) are estimated using the full covariance matrix. The cell e.s.d.'s are taken into account individually in the estimation of e.s.d.'s in distances, angles and torsion angles; correlations between e.s.d.'s in cell parameters are only used when they are defined by crystal symmetry. An approximate (isotropic) treatment of cell e.s.d.'s is used for estimating e.s.d.'s involving l.s. planes.
Refinement. Refinement of *F*^2^ against ALL reflections. The weighted *R*-factor *wR* and goodness of fit *S* are based on *F*^2^, conventional *R*-factors *R* are based on *F*, with *F* set to zero for negative *F*^2^. The threshold expression of *F*^2^ > σ(*F*^2^) is used only for calculating *R*-factors(gt) *etc*. and is not relevant to the choice of reflections for refinement. *R*-factors based on *F*^2^ are statistically about twice as large as those based on *F*, and *R*- factors based on ALL data will be even larger.

### Fractional atomic coordinates and isotropic or equivalent isotropic displacement parameters (Å^2^)

**Table:**

	*x*	*y*	*z*	*U*_iso_*/*U*_eq_	
O1	0.21682 (7)	0.58070 (13)	0.26522 (8)	0.0532 (4)	
O2	0.59318 (7)	0.69018 (13)	0.32421 (9)	0.0594 (4)	
H2O	0.6310 (12)	0.649 (2)	0.2968 (14)	0.089*	
O3	0.28796 (9)	0.08570 (16)	−0.06956 (10)	0.0762 (5)	
O4	0.13699 (9)	−0.00902 (16)	−0.07979 (10)	0.0708 (5)	
H4O	0.1798 (13)	−0.024 (3)	−0.1090 (16)	0.106*	
C1	0.43059 (10)	0.50808 (17)	0.22500 (11)	0.0431 (4)	
H1	0.4245	0.4397	0.1889	0.052*	
C2	0.50835 (10)	0.54733 (17)	0.24660 (11)	0.0445 (4)	
H2	0.5541	0.5065	0.2246	0.053*	
C3	0.51802 (10)	0.64693 (17)	0.30075 (11)	0.0423 (4)	
C4	0.44972 (11)	0.70803 (18)	0.33262 (12)	0.0511 (5)	
H4	0.4562	0.7759	0.3690	0.061*	
C5	0.37224 (10)	0.66855 (17)	0.31058 (11)	0.0461 (4)	
H5	0.3267	0.7097	0.3327	0.055*	
C6	0.36105 (9)	0.56830 (15)	0.25593 (10)	0.0366 (4)	
C7	0.27741 (10)	0.53112 (17)	0.23093 (10)	0.0400 (4)	
C8	0.26703 (10)	0.43695 (18)	0.16605 (11)	0.0453 (4)	
H8	0.3133	0.4132	0.1362	0.054*	
C9	0.19621 (11)	0.38287 (17)	0.14669 (11)	0.0459 (4)	
H9	0.1504	0.4111	0.1755	0.055*	
C10	0.18214 (10)	0.28457 (17)	0.08558 (11)	0.0444 (4)	
C11	0.10435 (11)	0.2317 (2)	0.07729 (12)	0.0523 (5)	
H11	0.0613	0.2625	0.1092	0.063*	
C12	0.09021 (12)	0.1337 (2)	0.02206 (12)	0.0570 (5)	
H12	0.0378	0.0990	0.0174	0.068*	
C13	0.15256 (12)	0.08717 (19)	−0.02586 (11)	0.0505 (5)	
C14	0.23128 (11)	0.13987 (18)	−0.01877 (12)	0.0496 (5)	
C15	0.24529 (11)	0.23674 (18)	0.03604 (11)	0.0494 (5)	
H15	0.2977	0.2714	0.0405	0.059*	
C16	0.37141 (14)	0.1250 (3)	−0.0603 (2)	0.1069 (11)	
H16A	0.4061	0.0719	−0.0937	0.160*	
H16B	0.3873	0.1169	−0.0038	0.160*	
H16C	0.3770	0.2128	−0.0772	0.160*	

### Atomic displacement parameters (Å^2^)

**Table:**

	*U*^11^	*U*^22^	*U*^33^	*U*^12^	*U*^13^	*U*^23^
O1	0.0284 (7)	0.0681 (9)	0.0632 (8)	0.0049 (6)	0.0022 (5)	−0.0101 (7)
O2	0.0294 (7)	0.0634 (9)	0.0855 (10)	−0.0054 (6)	−0.0028 (6)	−0.0207 (8)
O3	0.0523 (9)	0.0907 (12)	0.0856 (11)	−0.0047 (8)	−0.0006 (8)	−0.0401 (9)
O4	0.0661 (10)	0.0756 (10)	0.0707 (10)	−0.0206 (8)	−0.0097 (7)	−0.0194 (8)
C1	0.0338 (9)	0.0456 (10)	0.0500 (10)	0.0044 (7)	−0.0024 (7)	−0.0103 (8)
C2	0.0275 (9)	0.0510 (10)	0.0550 (10)	0.0064 (7)	0.0007 (7)	−0.0087 (9)
C3	0.0290 (9)	0.0447 (10)	0.0530 (10)	−0.0026 (7)	−0.0014 (7)	−0.0011 (8)
C4	0.0373 (10)	0.0531 (11)	0.0629 (12)	−0.0020 (8)	0.0042 (8)	−0.0216 (9)
C5	0.0310 (9)	0.0517 (10)	0.0555 (11)	0.0030 (8)	0.0083 (7)	−0.0108 (9)
C6	0.0287 (8)	0.0403 (9)	0.0407 (9)	0.0004 (7)	0.0005 (6)	0.0004 (8)
C7	0.0303 (9)	0.0444 (9)	0.0452 (9)	0.0018 (7)	0.0005 (7)	0.0033 (8)
C8	0.0307 (9)	0.0561 (11)	0.0491 (10)	0.0000 (8)	−0.0005 (7)	−0.0049 (9)
C9	0.0342 (10)	0.0514 (10)	0.0520 (10)	−0.0002 (8)	−0.0001 (8)	0.0006 (9)
C10	0.0347 (9)	0.0494 (10)	0.0490 (10)	−0.0053 (8)	−0.0055 (8)	0.0043 (8)
C11	0.0390 (10)	0.0655 (12)	0.0524 (11)	−0.0099 (9)	−0.0016 (8)	0.0033 (10)
C12	0.0441 (11)	0.0695 (13)	0.0575 (12)	−0.0221 (10)	−0.0100 (9)	0.0057 (10)
C13	0.0516 (12)	0.0526 (11)	0.0475 (10)	−0.0106 (9)	−0.0139 (9)	0.0030 (9)
C14	0.0426 (11)	0.0545 (11)	0.0518 (10)	−0.0028 (9)	−0.0076 (8)	−0.0008 (9)
C15	0.0361 (9)	0.0552 (11)	0.0568 (11)	−0.0071 (8)	−0.0065 (8)	−0.0035 (9)
C16	0.0457 (14)	0.139 (3)	0.136 (3)	−0.0060 (15)	0.0096 (14)	−0.076 (2)

### Geometric parameters (Å, º)

**Table:**

O1—C7	1.2461 (19)	C7—C8	1.453 (2)
O2—C3	1.359 (2)	C8—C9	1.322 (2)
O2—H2O	0.871 (16)	C8—H8	0.9300
O3—C14	1.362 (2)	C9—C10	1.448 (2)
O3—C16	1.427 (3)	C9—H9	0.9300
O4—C13	1.358 (2)	C10—C11	1.388 (2)
O4—H4O	0.857 (17)	C10—C15	1.399 (2)
C1—C2	1.376 (2)	C11—C12	1.381 (3)
C1—C6	1.389 (2)	C11—H11	0.9300
C1—H1	0.9300	C12—C13	1.370 (3)
C2—C3	1.372 (2)	C12—H12	0.9300
C2—H2	0.9300	C13—C14	1.399 (2)
C3—C4	1.383 (2)	C14—C15	1.368 (3)
C4—C5	1.375 (2)	C15—H15	0.9300
C4—H4	0.9300	C16—H16A	0.9600
C5—C6	1.386 (2)	C16—H16B	0.9600
C5—H5	0.9300	C16—H16C	0.9600
C6—C7	1.473 (2)		
			
C3—O2—H2O	109.3 (16)	C8—C9—C10	127.20 (17)
C14—O3—C16	117.46 (16)	C8—C9—H9	116.4
C13—O4—H4O	110 (2)	C10—C9—H9	116.4
C2—C1—C6	121.48 (16)	C11—C10—C15	118.20 (17)
C2—C1—H1	119.3	C11—C10—C9	119.54 (17)
C6—C1—H1	119.3	C15—C10—C9	122.21 (15)
C3—C2—C1	119.68 (16)	C12—C11—C10	120.65 (18)
C3—C2—H2	120.2	C12—C11—H11	119.7
C1—C2—H2	120.2	C10—C11—H11	119.7
O2—C3—C2	122.38 (15)	C13—C12—C11	120.68 (17)
O2—C3—C4	117.73 (16)	C13—C12—H12	119.7
C2—C3—C4	119.88 (15)	C11—C12—H12	119.7
C5—C4—C3	120.11 (17)	O4—C13—C12	119.51 (17)
C5—C4—H4	119.9	O4—C13—C14	120.99 (19)
C3—C4—H4	119.9	C12—C13—C14	119.50 (18)
C4—C5—C6	120.98 (16)	O3—C14—C15	126.17 (17)
C4—C5—H5	119.5	O3—C14—C13	114.04 (17)
C6—C5—H5	119.5	C15—C14—C13	119.79 (18)
C5—C6—C1	117.86 (15)	C14—C15—C10	121.17 (17)
C5—C6—C7	119.86 (15)	C14—C15—H15	119.4
C1—C6—C7	122.26 (15)	C10—C15—H15	119.4
O1—C7—C8	120.98 (15)	O3—C16—H16A	109.5
O1—C7—C6	119.90 (15)	O3—C16—H16B	109.5
C8—C7—C6	119.12 (14)	H16A—C16—H16B	109.5
C9—C8—C7	124.31 (16)	O3—C16—H16C	109.5
C9—C8—H8	117.8	H16A—C16—H16C	109.5
C7—C8—H8	117.8	H16B—C16—H16C	109.5
			
C6—C1—C2—C3	0.9 (3)	C8—C9—C10—C11	175.35 (19)
C1—C2—C3—O2	−179.82 (17)	C8—C9—C10—C15	−2.2 (3)
C1—C2—C3—C4	−0.7 (3)	C15—C10—C11—C12	0.6 (3)
O2—C3—C4—C5	179.66 (17)	C9—C10—C11—C12	−177.03 (17)
C2—C3—C4—C5	0.5 (3)	C10—C11—C12—C13	−0.3 (3)
C3—C4—C5—C6	−0.5 (3)	C11—C12—C13—O4	−179.87 (18)
C4—C5—C6—C1	0.8 (3)	C11—C12—C13—C14	−0.2 (3)
C4—C5—C6—C7	−177.68 (17)	C16—O3—C14—C15	5.9 (3)
C2—C1—C6—C5	−1.0 (3)	C16—O3—C14—C13	−173.8 (2)
C2—C1—C6—C7	177.44 (16)	O4—C13—C14—O3	−0.3 (3)
C5—C6—C7—O1	−7.4 (3)	C12—C13—C14—O3	−179.98 (18)
C1—C6—C7—O1	174.18 (16)	O4—C13—C14—C15	179.99 (17)
C5—C6—C7—C8	172.36 (16)	C12—C13—C14—C15	0.3 (3)
C1—C6—C7—C8	−6.0 (2)	O3—C14—C15—C10	−179.67 (18)
O1—C7—C8—C9	−10.4 (3)	C13—C14—C15—C10	0.0 (3)
C6—C7—C8—C9	169.82 (17)	C11—C10—C15—C14	−0.5 (3)
C7—C8—C9—C10	−176.97 (17)	C9—C10—C15—C14	177.08 (17)

### Hydrogen-bond geometry (Å, º)

Cg is the centroid of the C1–C6 benzene ring.

**Table:**

*D*—H···*A*	*D*—H	H···*A*	*D*···*A*	*D*—H···*A*
O4—H4*O*···O3	0.86 (2)	2.20 (3)	2.655 (2)	113 (2)
O2—H2*O*···O1^i^	0.87 (2)	1.87 (2)	2.7349 (18)	173 (2)
O4—H4*O*···O1^ii^	0.86 (2)	2.22 (2)	2.937 (2)	141 (3)
C16—H16*A*···*Cg*^ii^	0.96	2.86	3.747 (3)	155

Symmetry codes: (i) *x*+1/2, *y*, −*z*+1/2; (ii) *x*, −*y*+1/2, *z*−1/2.

## References

[bb1] Allen, F. H., Kennard, O., Watson, D. G., Brammer, L., Orpen, A. G. & Taylor, R. (1987). *J. Chem. Soc. Perkin Trans. 2*, pp. S1–19.

[bb2] Altomare, A., Cascarano, G., Giacovazzo, C. & Guagliardi, A. (1993). *J. Appl. Cryst.* **26**, 343–350.

[bb3] Bruker (2004). *APEX2*, *SAINT*, *XPREP* and *SADABS* Bruker AXS Inc., Madison, Wisconsin, USA.

[bb4] Chitra, M., Jonathan, D. R., Rajan, Y. C. & Duraipandiyan, V. (2013). *Int. J. Chem. Appl.* **5**, 73–81.

[bb5] Di Carlo, G., Mascolo, N., Izzo, A. A. & Capasso, F. (1999). *Life Sci.* **65**, 337–353. 10.1016/s0024-3205(99)00120-4 10421421

[bb6] Farrugia, L. J. (2012). *J. Appl. Cryst.* **45**, 849–854.

[bb7] Jasinski, J. P., Butcher, R. J., Musthafa Khaleel, V., Sarojini, B. K. & Narayana, B. (2011). *Acta Cryst* E**67**, o813. 10.1107/S1600536811007781 PMC3099842 21754099

[bb8] Marais, J. P. J., Ferreira, D. & Slade, D. (2005). *Phytochemistry*, **66**, 2145–2176. 10.1016/j.phytochem.2005.03.006 16153413

[bb9] Ni, L., Meng, C. Q. & Sikorski, J. A. (2004). *Exp. Opin. Ther. Pat.* **14**, 1669–1691.

[bb10] Sheldrick, G. M. (2008). *Acta Cryst.* A**64**, 112–122. 10.1107/S0108767307043930 18156677

[bb11] Sidharthan, J., Jonathan, D. R. & Amaladhas, T. P. (2012). *Int. J. Chem. Appl.* **4**, 241–250.

[bb12] Spek, A. L. (2009). *Acta Cryst.* D**65**, 148–155. 10.1107/S090744490804362X PMC2631630 19171970

[bb13] Troeberg, L., Chen, X., Flaherty, T. M., Morty, R. E., Cheng, M., Springer, H. C., McKerrow, J. H., Kenyon, G. L., Lonsdale-Eccles, J. D., Coetzer, T. H. T. & Cohen, F. E. (2000). *Mol. Med.* **6**, 660–669. PMC1949976 11055585

